# Pleiotrophin-Loaded Mesoporous Silica Nanoparticles as a Possible Treatment for Osteoporosis

**DOI:** 10.3390/pharmaceutics15020658

**Published:** 2023-02-16

**Authors:** Daniel Lozano, Beatriz Leiva, Inés S. Gómez-Escalonilla, Sergio Portal-Núñez, Arancha R. de Górtazar, Miguel Manzano, María Vallet-Regí

**Affiliations:** 1 Departamento de Química en Ciencias Farmacéuticas, Facultad de Farmacia, Universidad Complutense de Madrid, Instituto de Investigación Hospital 12 de Octubre (i+12), Plaza de Ramón y Cajal s/n, 28040 Madrid, Spain; 2 Centro de Investigación Biomédica en Red de Bioingeniería, Biomateriales y Nanomedicina (CIBER-BBN), 28029 Madrid, Spain; 3 Grupo de Fisiopatología Ósea, Departamento de Ciencias Médicas Básicas, Instituto de Medicina Aplicada de la Universidad San Pablo-CEU, Facultad de Medicina, Universidad San Pablo CEU, CEU Universities, Urbanización Montepríncipe s/n, 28925 Madrid, Spain

**Keywords:** nanoparticles, mesoporous materials, pleiotrophin, osteoporosis, osteoblasts, stem cells, polyethylenimine, osteogenic

## Abstract

Osteoporosis is the most common type of bone disease. Conventional treatments are based on the use of antiresorptive drugs and/or anabolic agents. However, these treatments have certain limitations, such as a lack of bioavailability or toxicity in non-specific tissues. In this regard, pleiotrophin (PTN) is a protein with potent mitogenic, angiogenic, and chemotactic activity, with implications in tissue repair. On the other hand, mesoporous silica nanoparticles (MSNs) have proven to be an effective inorganic drug-delivery system for biomedical applications. In addition, the surface anchoring of cationic polymers, such as polyethylenimine (PEI), allows for greater cell internalization, increasing treatment efficacy. In order to load and release the PTN to improve its effectiveness, MSNs were successfully internalized in MC3T3-E1 mouse pre-osteoblastic cells and human mesenchymal stem cells. PTN-loaded MSNs significantly increased the viability, mineralization, and gene expression of alkaline phosphatase and Runx2 in comparison with the PTN alone in both cell lines, evidencing its positive effect on osteogenesis and osteoblast differentiation. This proof of concept demonstrates that MSN can take up and release PTN, developing a potent osteogenic and differentiating action in vitro in the absence of an osteogenic differentiation-promoting medium, presenting itself as a possible treatment to improve bone-regeneration and osteoporosis scenarios.

## 1. Introduction

The current population is ageing because life expectancy has increased in the last decades [1]. As a consequence, the impact of bone diseases on society is increasing, especially in postmenopausal women and in the elderly [1,2]. In this context, osteoporosis is becoming a more common disease in the ageing population with altered bone remodeling, where the osteoblasts (bone-forming cells) and osteoclasts (bone-resorbing cells) work in an uncoordinated process [3]. The lack of cellular communication leads to the typical loss of bone mass together with the deterioration of the microarchitecture of bone tissue and the consequent risk of fracture. It affects one in three women and one in five men according to the International Osteoporosis Foundation (IOF) and is associated with more than nine million fractures worldwide [4]. As a result, the disease has both a real human and socio-economic impact.

Current treatments for osteoporosis are based on the use of antiresorptive drugs, which inhibit the action of osteoclasts, and/or anabolic agents that activate the action of osteoblasts. These include bisphosphonates (Alendronate, Ibandronate...), monoclonal antibodies (Denosumab or Romosozumab), or hormones and proteins such as parathormone (PTH) [5]. Other proteins, such as PTH-related protein (PTHrP), osteostatin, bone morphogenic proteins (BMPs), or growth factors, are under study [6,7]. However, conventional treatments have certain limitations, either due to the lack of the bioavailability of these therapeutic agents in bone tissue or the toxicity in non-specific tissues [8,9]. In addition, many of the treatments are no longer active after two years [5,6,7,8,9]. Therefore, new and more effective treatments are being explored, especially considering the increasing incidence of osteoporosis expected in the next few years.

In recent years, the search for new molecules for the treatment of osteoporosis has been intensified. Among them include osteogenic macromolecules such as pleiotrophin (PTN), a potent cytokine that binds to proteoglycans and glycosaminoglycans, such as chontroitin sulphate (CS), to receptors, such as tyrosine phosphatase beta/zeta (RPTPβ/ζ), and plays a major role in bone remodeling [10,11]. PTN is a cationic protein that presents potent mitogenic, angiogenic, and chemogenetic activity [12,13]. Consequently, its name derives from its pleiotropic nature as it exerts very different actions depending on the stimulus received [14,15]. It has been associated with a wide range of important biological events, including neural regeneration, bone development, inflammation, cancer metastasis, and tissue repair [16,17]. However, if PTN is to be used as a growth factor in bone tissues, its local administration as an osteogenic ectopic growth factor might be considered [18,19]. In this sense, using nanoparticles to deliver PTN to specific bone tissues could be a smart alternative to obtain high concentrations of PTN in those tissues.

Among all types of nanoparticles, mesoporous silica nanoparticles (MSNs) have emerged as excellent drug carriers [20,21]. In fact, they have been proposed as inorganic drug-delivery systems (DDSs) for various applications and approaches in biomedicine [22,23]. These MSNs exhibited unique properties as a spherical shape, physicochemical stability, and modulable size depending on their synthesis and, more importantly, a great loading capacity for different types of cargo [24,25,26,27,28,29,30]. Due to their textural properties of a high surface area and pore volume, they can host a variety of therapeutic agents, such as antibiotics, biomolecules, or peptides such as osteostatin (OST) [31,32], and can even carry gene-transfection agents (siRNA, miRNA) [31,32,33,34]. The use of these nanocarriers has been studied extensively in different types of cells where their low cytotoxicity, internalization, and long retention time have been demonstrated [26,35,36]. In addition, coating with cationic polymers, such as polyethylenimine (PEI), allows for a greater internalization of MSNs and endosomal escape by the proton-sponge effect [35].

**Scheme 1:**
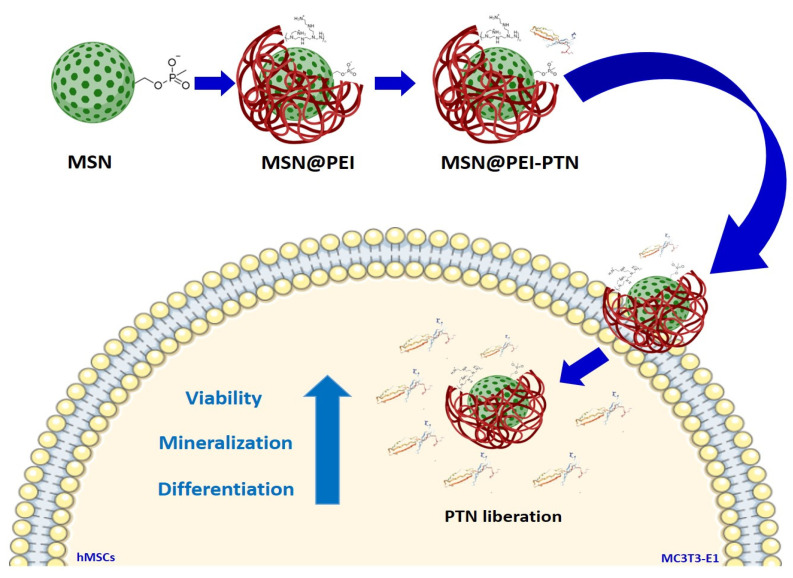
Illustration of the preparation, PEI pre-coating, pleiotrophin (PTN) loading, and intracellular release of MSN@PEI-PTN nanocarriers and their effects in human Mesenchymal Stem Cells (hMSCs) and mouse pre-osteoblastic MC3T3-E1 cells.

The overall objective of this work was to develop and evaluate a nanocarrier that is based on MSNs with PEI pre-coating loaded with PTN (Scheme 1). This novel platform has been evaluated here in two bone-related cell lines with very promising results. The internalization, viability, mineralization, and differentiation results showed in this study envision a potential new treatment for osteoporosis that may be more effective than current treatments.

## 2. Materials and Methods

### 2.1. Synthesis of Mesoporous Silica Nanoparticles

The synthesis of MCM-41 type MSNs was carried out using a modified Stöber method [37]. Briefly, 0.6 g of cetyl-trimethylammonium bromide (CTAB) and 300 mL of a 0.32 M ammonium hydroxide solution was added to in a 500 mL beaker. The mixture was carried out at 50 °C for 1 h, under moderate stirring. Then, 6 mL of a 1:4 ratio of tetraethyl orthosilicate (TEOS) and ethanol solution was added and left to stand overnight. Then, the suspension mixture was pipetted, and the precipitate formed was discarded. The suspension was subjected to a hydrothermal-ageing treatment at 70 °C in a closed bottle overnight, and the solid was separated by centrifugation. The surfactant used as a template for the mesoporous structure was removed by an ion-exchange extraction process, with an extraction NH_4_NO_3_ (10 mg/mL) solution in ethanol (95%) for two cycles, to ensure maximum surfactant extraction. These MSNs were synthesized by a co-condensation of TEOS with a functional organosilane 3-trihydroxysilylpropyl methylphosphonate TSPMP (50%w, 0.14 mL, 0.37 mmol). In addition, MSNs were labelled by a co-condensation of amino propyltriethoxysilane APTES (98%) with Rhodamine-B or fluorescein isocyanate (FITC, 1.54 × 10^−3^ mmol).

### 2.2. Poly(ethylenimine) Grafting

To perform the polymer coating, 5 mg of phosphonate-modified MSNs were dispersed in 1 mL of absolute ethanol with a (2:1) MSN@PEI (5KDa) ratio (2:1), shown in previous studies to be non-toxic [31,34,36]. Although some cytotoxicity has been reportedly associated with this type of polymer, below a certain molecular weight, it is perfectly biocompatible [31,32,36]. The mixture, well-dispersed in ultrasound, was stirred for 30 min at room temperature and then washed with phosphate-buffered saline and ethanol. All reagents used for the synthesis of MSNs and their coating were commercial products from Merck (Spain) and were used without further purification.

### 2.3. Pleiotrophin Loading

The recombinant human-protein Pleiotrophin (R&D Systems™) was carried out in a 1:4–10^−^^4^ MSN@PEI-PTN ratio, where, for each mg of MSN@PEI, there would be 0.4 μg of PTN (0.05 nM).

### 2.4. Physicochemical Characterization of MSNs

The MSNs were characterized by electronic microscopy and chemical- and surface-characterization techniques. The micrographs were obtained by transmission-electron microscopy (TEM) in the JEOL JEM 2100 equipment at 200 kV, which was equipped with a KeenView camera. Particle size was determined by Dynamic Light Scattering (DLS) analyzed together with Z-potential (ζ) on a Zetasizer Nano ZS (Malvern Instruments, Westborough, MA, USA) equipped with a 633 nm red laser, which was measured in a disposable plastic cell in water at room temperature. For the analysis of surface and textural characteristics, a Micromeritics 3 Flex (Norcross, GA, USA) was used. For these measurements, the MSN samples were previously degassed for 24 h at 120 °C under vacuum. Surface area (S_BET_) and pore volume (Vp) were calculated using the Brunauer-Emmett-Teller (BET) method, and pore-size distributions were obtained using a Density Functional Theory (DFT) method and a hydroxylated cylindrical pore-surface model. According to the literature, the accuracy of the DFT method is higher than with BJH theory and offers a more reliable fit at pore sizes smaller than 20 nm, with an ability to detect the microporosity of the structure. Diffractograms were obtained by small-angle X-ray diffraction (SA-XRD) on a Philips X-Pert MPD diffractometer equipped with Cu–Kα radiation. This technique revealed the arrangement of the mesostructure of the pores inside the MSNs, analyzed in a low-angle range of 2θ (0.5–10°). Thermogravimetric analysis of MSNs was carried out to measure extraction efficiency, quantify the degree of MSN@PEI coating, and assess PTN-protein loading, which was carried out by quantifying the mass loss in a fixed range between 150 and 600 °C. The data obtained using a PerkinElmer Pyris Diamond TG/DTA were obtained with increasing temperature conditions from 5 °C/min up to 600 °C. Chemical characterizations by Fourier-transform infrared spectroscopy (FTIR) were carried out using a Nicolet Nexus spectrometer (Thermo Fisher Scientific, Waltham, MA, USA) that was equipped with a SMART Golden Gate diffuse reflectance accessory for attenuated total reflection (ATR) analysis.

### 2.5. Cell Cultures

#### 2.5.1. Cell Types

Cell assays were performed using MC3T3-E1 mice pre-osteoblastic cells (ECACC 99072810) and human mesenchymal stem cells (hMSCs) isolated from normal (non-diabetic) adult human bone marrow extracted from bilateral punctures of the posterior iliac crests of healthy volunteers (Lonza, PT-2501, Batch Nº: 19TL16885) [38,39]. The cells were maintained at 37 °C, 5% CO_2_, and 95% humidity in alfa MEM medium (supplemented with 10% FBS, L-glutamine, and antibiotic) and specialized mesenchymal stem-cell culture medium (supplemented with SingleQuots^®^(Poietics^TM^)), respectively. The cells used at passages #5–6 were seeded for assays at a density of 2.5 × 10^4^ cells/cm^2^. When they reached 40–60% confluence, they were incubated for 2 h with the MSNs to study their behavior inside the cells, and the time was optimized from previous studies with this cell types [38]. Each experiment was performed in triplicate, and representative results were shown per group tested. Cell counting was performed using trypan-blue staining (0.4%, Invitrogen™), which quantifies membrane-compromised cells, in a Countess^TM^ cell counter where cell number and viability are measured by exclusion on a disposable slide with a cell-counting chamber.

#### 2.5.2. Viability and Cytotoxicity

Cytotoxicity was measured by a quantification of viability at 24/48 h using Alamar Blue^®^ reagent (Invitrogen™, Groningen, The Netherlands) (according to the manufacturer’s instructions) in both cell types with different concentrations of MSN and MSN@PEI (25–50–100–200 μg/mL) in the presence or absence of PTN [36]. Cellular metabolic activity was quantified by the conversion of resazurin to resorufin by measuring its fluorescence at λ_EX_560 nm/λ_EM_590 nm on a Synergy 2 luminescence multimode microplate reader (BioTek^®^, Winooski, VT, USA) after 2 h of cell internalization.

#### 2.5.3. Cell Uptake

Flow-cytometry measurements were performed using flow cytometry (IS conditions) on a FACScan (Becton, Dickinson and Company, Mountain View, CA, USA) at an excitation wavelength of 488 nm, measuring at 530 nm, and the activator was set to the green fluorescence channel (FL1) [40]. For statistical significance, at least 10,000 cells in each sample were analyzed, and the mean fluorescence emitted by cells that took up the fluorescein-labelled MSN@PEI nanocarrier was used (25, 50 and 100 μg/mL). Trypan blue was used to eliminate extracellular fluorescence. Images obtained to confirm internalization were taken using an optical microscope at 20× magnification.

#### 2.5.4. Fluorescence Microscopy

Micrographs to check the internalization of the different nanosystems were obtained using an Evos FL Cell Imaging System microscope equipped with three light lasers (DAPI (357/44; 447/60), GFP (470/22; 525/50), and RFP (531/40; 593/40). Cells were fixed with p-formaldehyde (45% *w*/*v*) and stained with phalloidin, (Atto 565) used to detect the cytoskeleton, and Fluoroshield™ with DAPI, used to stain cell nuclei. All reagents were obtained from Merck, Spain.

#### 2.5.5. Mineralization

Osteoblastic-mineralization studies were carried out by performing an alizarin-red staining after 15 days. Calcium deposits formed after treating cells for 2 h with 0.05 nM PTN and after 100 μg/mL MSN@PEI-PTN were quantified. In the present mineralization assay, alizarin-red reagent (0.136 g, 40 mM pH 4.2) was used as a dye with an affinity to the calcium ion, an ion secreted by hMSCs upon differentiation [38]. This dye was eluted with 10% *p*/*v* cetylperidinium chloride reagent in 10 mM sodium phosphate buffer of pH 7. All reagents were obtained from Merck, Spain. Finally, absorbance was measured at 620 nm in a Synergy 2 multimode luminescence microplate reader (BioTek^®^) to quantify the mineralization produced.

#### 2.5.6. Gene Expression

Both cell lines were cultured for 5 days in the presence or absence of different nanosystems for differential gene-expression studies. The gene expression of the osteoblastic-differentiation markers, Alkaline Phosphatase (ALP) and Runx2, were quantified using real-time PCR, QuantStudio5 equipment, and a previously described protocol (Applied Biosystem-Thermo Scientific, Foster City, CA, USA) [36]. TaqMan^MGB^ probes were obtained using Assay-by-Design^SM^ (Applied Biosystems). For each sample and using the given cycle-threshold (Ct) value, mRNA copy numbers were calculated and glyceraldehyde-3-phosphate dehydrogenase (GAPDH) rRNA (a housekeeping gene) was amplified in parallel with the tested genes. The number of amplification steps required to reach an arbitrary Ct was computed. The relative gene expression was represented by 2^−ΔΔCt^, where ΔΔCt = ΔC_target gene_ − ΔCt_GAPDH_. The fold change for the treatment was defined as the relative expression compared to the control GAPDH expression, calculated as 2^−ΔΔCt^, where ΔΔCt = ΔC_treatment_ − ΔC_control_.

#### 2.5.7. Statistical Analysis

The results presented below are expressed as mean ± standard error of the mean (SEM). Statistical analyses of differences between groups were performed using the Student’s *t*-test. All values of *p* < 0.05 were considered significant.

## 3. Results and Discussion

The limitations in the current treatments of osteoporosis have made it necessary to search for different alternatives [6,7]. In this sense, nanotechnology might provide advanced alternatives to solve these problems [24,26,27,28,31]. To this end, a novel nanosystem based on MSNs loaded with a pleiotrophin osteogenic peptide and coated with PEI polymer, which improves the internalization of the nanocarrier, has been developed and evaluated here in two bone-related cell lines. Internalization, viability, mineralization, and differentiation gene-expression studies were performed to show the efficacy of this nanosystem in mouse pre-osteoblastic MC3T3-E1 cells and human Mesenchymal Stem Cells (hMSC) isolated from normal adult human bone marrow extracted from bilateral punctures of the posterior iliac crests of healthy volunteers [38,39].

### 3.1. Synthesis and Physico-Chemical Characterization of Nanosystems

**Figure 1:**
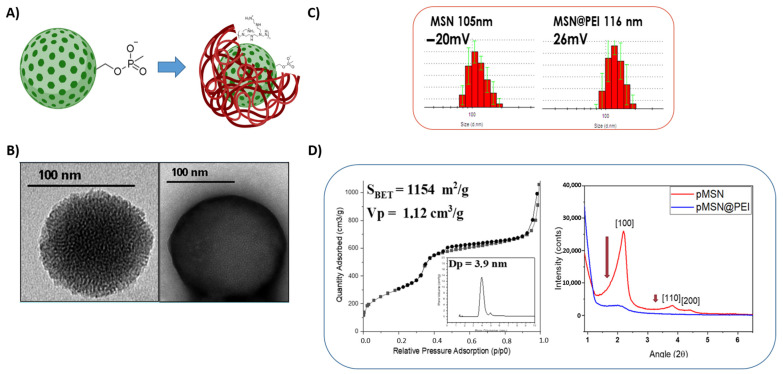
Synthesis and characterization of MSN and MSN@PEI nanosystems. (**A**) Schematic representation of the designed nanocarrier based on mesoporous silica nanoparticles with and without PEI. (**B**) TEM micrographs of the nanoparticles before and after coating with PEI polymer. (**C**) The ζ potential and DLS analysis, (**D**) N_2_ adsorption (BET), and X-ray diffraction (XRD) before and after coating with PEI polymer.

Mesoporous silica nanoparticles were produced following a modification of the Stöber method in which a surfactant was employed as a structure-directing agent to produce the mesostructured silica. Nanoparticles with mesoporous structure and a spherical shape of ca. 80–120 nm in diameter, measured by DLS, were obtained. The MSNs were coated with PEI to ensure the PTN loading and favor cellular internalization (Figure 1A).

The correct synthesis of MSNs and the subsequent modification of their surface were confirmed through several characterization techniques. Transmission-electron microscopy of the MSNs revealed the small size and mesoporous structure of the particles (Figure 1B). The surface properties of the MSNs were analyzed using a N_2_ isotherm according to a Brunauer-Emmett-Teller (BET) model with an area of 1.150 ± 10 m^2^/g, a pore volume of 1.12 cm^3^/g, and a pore size that was adjusted to 3.94 nm (Figure 1D), and obtained by a method based on density functional theory (DFT). The diffraction maxima corresponding to the 100, 110, and 200 planes were analyzed, confirming the hexagonal mesostructured of the pores, which is typical of MCM-41 materials (Figure 1) [20,21,22,23]. These data confirm that the external surface of the particles was successfully grafted with the polymer.

**Figure 2:**
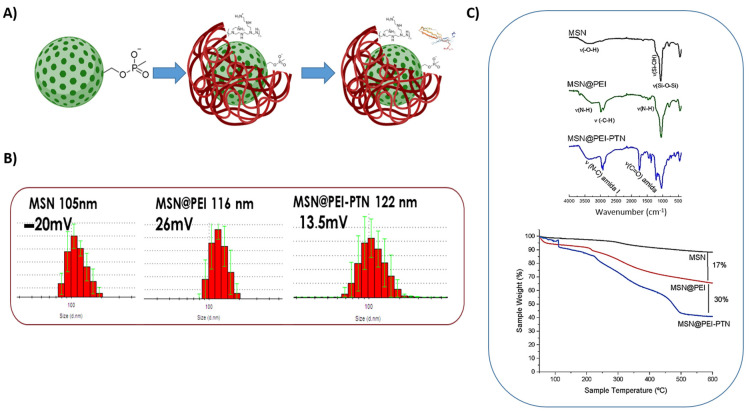
Synthesis and characterization of MSN@PEI-PTN nanosystems. (**A**) Schematic representation of the designed nanocarrier based on mesoporous silica nanoparticles with and without PEI and pleiotrophin (PTN). (**B**) The ζ potential and DLS analysis. (**C**) Fourier Transformed Infrared spectroscopy (FTIR) and thermogravimetric analysis (TGA) of MSN@PEI before and after loading with PTN peptide.

The nanoparticles were functionalized with phosphonate groups, providing the strong negative charge needed to coat them afterwards with the cationic PEI polymer. The functionalization of the nanoparticles with PEI was confirmed by several characterization techniques. In this regard, the correct polymer coating, which was not present on unmodified MSNs, was confirmed through the phosphotungstic-acid-stained layer covering the surface of PEI-functionalized nanoparticles (MSN@PEI), and it can be appreciated by TEM in Figure 1B. Additionally, FTIR spectroscopy showed the typical vibration bands from silica in MSNs (Figure 2): a broad vibration band in the 3000–3400 cm^−1^ region (O–H stretching vibration bands from the silanol groups, Si–OH); the Si–O in-plane stretching vibrations at ca. 950 cm^−1^ and the Si–O stretching vibrations; the intense Si–O covalent bond vibrations in the range of 1200–1000 cm^−1^; and the Si–O–Si symmetric stretching vibrations at ca. 800 cm^−1^ and its bending vibrations at ca. 470 cm^−1^. The functionalization of the MSNs with PEI was confirmed by the presence of several bands at ca. 2850–2990 cm^−1^, corresponding to the C–H asymmetric and symmetric stretching vibrations of the alkyl chains from PEI. Additionally, the vibration bands at ca. 3150 cm^−1^ (N–H stretching), ca. 1530 cm^−1^ (N–H deformation), and ca. 1400 cm^−1^ (C–N stretching) confirmed the correct grafting of PEI onto the surface of MSNs.

The Z-potential measurements of the particles dispersed in water confirmed the successful functionalization of MSNs with PEI. As previously mentioned, the MSNs were initially functionalized with phosphonate groups, inducing a higher negative charge (−20 mV) than MSNs without phosphonate groups (−5 mV), which are required to efficiently coat with the polymer. In this sense, there was a severe change from negative phosphonate functionalized MSNs (−20 mV) to positive (+26 mV) in PEI-coated MSNs (Figure 1 and Figure 2). The hydrodynamic size of the MSNs was evaluated using dynamic light scattering, showing that their monodispersity was unaffected by the functionalizing process. The amount of PEI grafted onto the MSN surface was quantified using thermal analyses, as observed in Figure 2. All these data confirmed that the external surface of the MSNs was successfully grafted with the PEI polymer.

Recombinant human-protein pleiotrophin (PTN) loading was carried out in a 1:4–10^−4^ MSN@PEI-PTN ratio, where, for each mg of MSN@PEI, there would be 0.4 μg of PTN. The protein loading was confirmed using FTIR to confirm the presence of typical C=O vibration bands from the PTN (Figure 2), in addition to the DLS results, where a slight increase of the hydrodynamic size was observed because of the PTN loading to the surface of the nanoparticles and the Z-potential values, which shifted from +26 mV of unloaded carriers to 13.5 mV in PTN-loaded nanoparticles. The charge reduction might be ascribed to the biomolecule presence on the surface of the developed MSNs. Moreover, the thermogravimetric analysis quantified 17% by the weight of PEI decorating the surface of the nanoparticles, and 30% of PTN proteins loaded in the nanocarrier system.

### 3.2. In Vitro Evaluation of Nanosystems

Previous results of our team and other laboratories have shown that MSNs can be functionalized and loaded with different biomolecules [22,23,24,25,26,27,28,29,30,31,32,33,34], including osteogenic peptides such as osteostatin, siRNAs, or miRNAs, which induce osteoblast proliferation and differentiation in vitro and in vivo in osteoblastic-impaired or osteoporosis scenarios [31,32].

In the present study, we loaded MSN@PEI with PTN in an attempt to develop an alternative treatment for osteoporosis by improving bone regeneration and increasing bone-cell viability, mineralization, and differentiation. This research is a proof-of-concept for this novel platform. In this sense, we have preliminarily addressed this hypothesis using an in vitro study with two bone-related type cells, MC3T3-E1 cells and hMSCs.

#### 3.2.1. Mouse Pre-Osteoblastic MC3T3-E1 Cells

**Figure 3:**
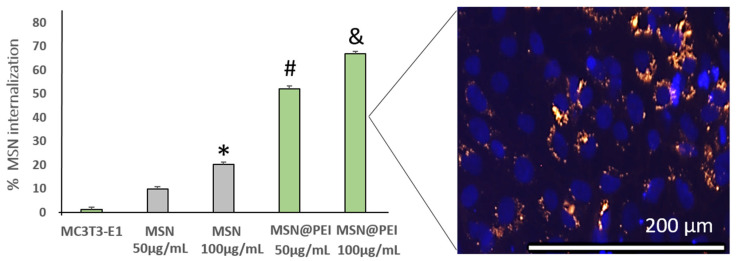
MSN-nanosystem internalization in mouse pre-osteoblastic MC3T3-E1 cells by flow cytometry and fluorescence microscopy. Cellular uptake of different Rhodamine-B-labeled MSNs and MSN@PEI was measured at 50 µg/mL and 100 µg/mL by flow cytometry at 2 h of internalization in MC3T3-E1 cells. Data are mean ± SEM of three independent experiments performed in triplicate. * *p* < 0.05 vs. MSN 50 µg/mL; # *p* < 0.01 vs. MSNs; & *p* < 0.05 vs. all groups. Representative images are MC3T3-E1 cells incubated with Rhodamine-B-labeled MSN@PEI at 100 µg/mL. Blue fluorescence (nuclei) and red fluorescence (Rh-MSN@PEI).

The first in vitro study to be carried out analyzed the cell uptake of nanosystems, with and without PEI coating, in order to find out whether the presence of the polymer could increase internalization after 2 h of MSN incubation. Flow-cytometry studies showed a significant increase in the internalization rate in mouse pre-osteoblastic MC3T3-E1 cells in a dose-dependant manner in both MSN and MSN@PEI nanosystems (Figure 3), with MSN@PEI at 100 µg/mL being the most effective. As shown in Figure 3, Rhodamine-B-labeled MSN@PEI (in red) were found in the cytoplasm, mostly around the nucleus that was stained blue with DAPI. This perinuclear location might be explained by the positive charge of the loaded MSNs (Figure 2B) and the negatively charged membrane of the nuclei. Furthermore, as discussed above, the present internalization results agree that coating with cationic polymers such as PEI increased the internalization of MSNs and endosomal escape due to the proton-sponge effect [31,32,36]

**Figure 4:**
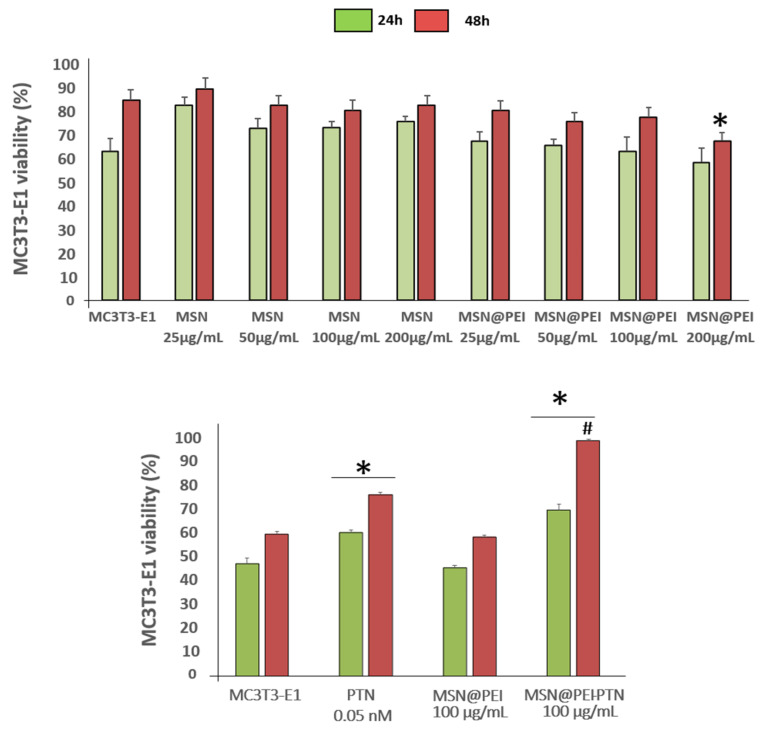
Mouse pre-osteoblastic MC3T3-E1 cell viability (measured by Alamar Blue^®^) in contact with different concentrations of MSN and MSN@PEI loaded or not with pleiotrophin (PTN) or in the presence of 0.05 nM exogenous PTN alone at 24 and 48 h of cell culture. Data are mean ± SEM of three independent experiments performed in triplicate. * *p* < 0.05 vs. MC3T3-E1 cells and MSN@PEI 100 µg/mL; # *p* < 0.01 vs. PTN 0.05 nM and all groups.

The next step in the biological evaluation of this nanocarrier was to show that none of the nanocarriers are toxic in the presence of different cell cultures. As is well-known, some cationic polymers, including PEI, can be toxic in different cell lines. The PEI used in this work was 5KD, which has been previously demonstrated to be non-toxic by Maria Vallet’s research group and other groups [33,34,36,40]. In this sense, viability results in mouse pre-osteoblastic cells (measured by Alamar Blue^®^) indicated that none of the nanosystems studied the affected cell viability at 24 and 48 h of cell culture, in the presence or absence of PEI polymer (Figure 4). The increase of the nanocarrier concentration slightly reduced the viability, as previously shown in the literature [31,32,40].

After internalization and cytotoxic evaluation of the here-developed nanoplatform, a concentration of 100 µg/mL of the MSN@PEI nanosystem was selected as the optimal concentration for the rest of the experiments. Very interesting results were observed when evaluating PTN-loaded MSNs with these cells. MSN@PEI-PTN nanocarrier significantly increased viability in comparison with free PTN at a 0.05 nM concentration, especially at 24 h, confirming the lack of cytotoxicity of the nanocarrier here-developed for the first time (Figure 4). These results demonstrate that PTN can be efficiently loaded and released from MSNs, producing an intense increase in bone-related cell viability even higher than that of free PTN. This is an important point since, as a growth factor in bone tissue, PTN must be administered in a localized manner as a part of ectopic osteogenic growth. The encapsulation of this biomolecule in the nanoplatform here-developed would ensure the local delivery of the PTN where it would produce a beneficial effect in the bone-tissue environment. Once there, the PTN release would increase bone-related cell viability and, therefore, bone regeneration.

In addition to improving the viability of pre-osteoblasts, the present nanosystems also significantly increase the mineralization and differentiation of these cells in carrying out their reparative function in situations of a loss of bone mass or function. For this reason, is important to study whether the complete MSN@PEI nanosystem loaded with PTN is capable of increasing calcium deposits (protein) and the gene expression of classic bone markers involved in osteoblast differentiation. It is important to note that the experiments carried out were performed in the absence of an osteogenic differentiation-promoting medium, which gives even more validity to the results obtained in this study related to osteoblastic differentiation and mineralization. Most studies involving the measurement of these parameters used β-glycerolphosphate and ascorbic acid to force the mineralization to more differentiated phenotypes, either in presence or absence of different types of biomaterials [41,42,43].

**Figure 5:**
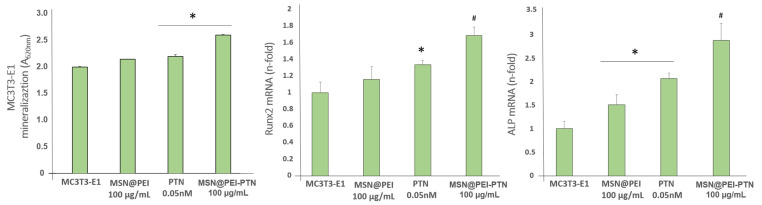
Mineralization and changes in osteoblastic gene expression in mouse pre-osteoblastic MC3T3-E1 cells induced by different nanosystems loaded or not with pleiotrophin (PTN) or in the presence of 0.05 nM exogenous PTN alone. Mineralization was assessed using alizarin-red staining at day 15. The gene expression of Runx2 and ALP markers (analyzed by real time PCR) was measured at day 5. Data are mean ± SEM of three independent experiments performed in triplicate. * *p* < 0.05 vs. MSN 100 µg/mL; # *p* < 0.01 vs. PTN 0.05 nM and all groups.

The differentiation assays revealed an increase in the mineralization of cells exposed to MSN@PEI compared to free PTN (Figure 5), confirming that the designed MSN@PEI-PTN system was effective for increasing the calcium deposits and could improve bioavailability in bone tissue. Although both PTN alone and PTN loaded into the nanocarrier significantly increased mineralization in these cells, which is also positive for the reasons mentioned above, no significant differences were observed between them. Besides the positive effect of our nanoparticles on osteoblast differentiation, we also evaluated the expression of two genes, Runx2 and ALP, that are indicative of an early bone-regeneration process. In this sense, the complete nanocarrier with PEI and PTN significantly increased the expression of Runx2 and ALP over the values of PTN. Again, the use of MSNs to deliver PTN has demonstrated better results than free PTN in triggering the bone-regeneration process through the expression of these genes. Although both free PTN and PTN loaded into the nanocarrier significantly increased the mineralization of these cells, the real effect was observed in the case of gene expression, where the complete nanosystem with PEI and PTN significantly increased the expression of Runx2 and ALP over the values of free PTN. These results support previous data regarding the use of PTN in a biomimetic scaffold [44].

#### 3.2.2. Human Mesenchymal Stem Cells (hMSCs)

Based on the above results, which demonstrated a positive effect of PTN loaded into the MSN@PEI nanosystem on the viability, mineralization, and differentiation of mouse pre-osteoblastic cells, an additional human cell line was selected to confirm these positive findings. Therefore, similar studies were carried out with human bone marrow-derived mesenchymal cells. Those cells were selected for the present study because of their human source and because they are known to play an important role in bone-regeneration processes [38].

**Figure 6:**
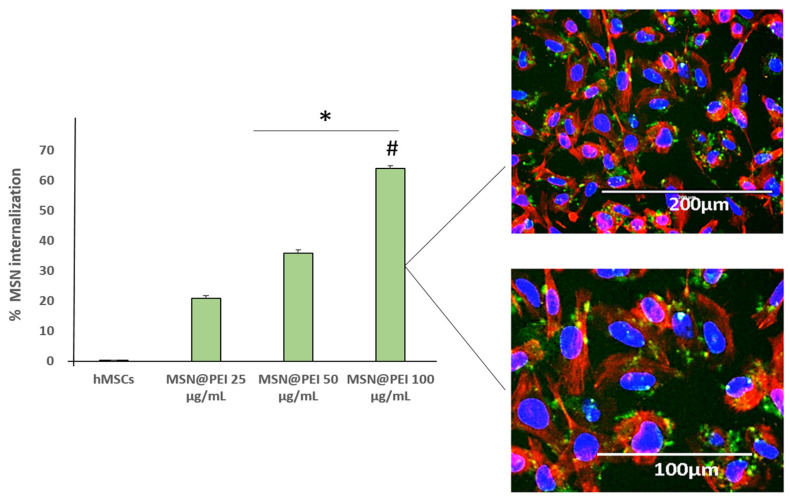
MSN-nanosystem internalization by human Mesenchymal Stem Cells (hMSC) using flow cytometry and fluorescence microscopy. The cellular uptake of different Fluorescein-labeled MSNs and MSN@PEI was measured at 25, 50, and 100 µg/mL by flow cytometry at 2 h of internalization in hMSCs. Data are mean ± SEM of three independent experiments performed in triplicate. * *p* < 0.05 vs. MSN 25 µg/mL; # *p* < 0.01 vs. all groups. Representative images at two magnifications of hMSCs incubated with Fluorescein-labeled MSN@PEI at 100 µg/mL at 2 h of internalization. Blue fluorescence (nuclei), red fluorescence (cytoplasm), and green (Fluorescein labeled MSN@PEI).

The obtained cell-internalization results were in agreement with those obtained for MC3T3-E1 cells. Cell-uptake studies by flow cytometry indicated a significant increase in the internalization rate in hMSCs in a dose-dependent manner in the presence of MSN@PEI nanosystems (Figure 6) at 25, 50, and 100 µg/mL concentrations, with MSN@PEI at 100 µg/mL being the most effective. In this sense, Figure 6 shows how Fluorescein-labeled MSN@PEI (in green) are located in the cytoplasms (in red) of hMSCs, as initially hypothesized here.

**Figure 7:**
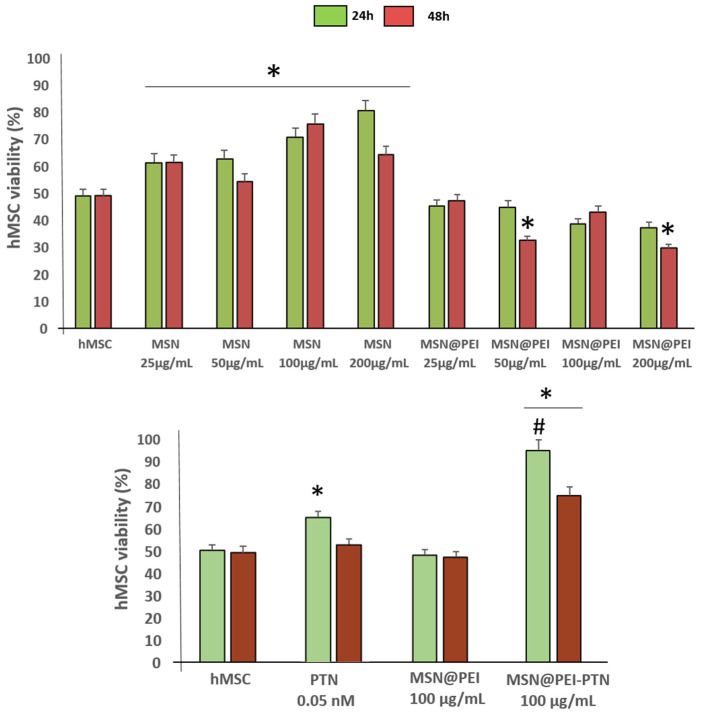
Human Mesenchymal Stem Cell (hMSC) viability (measured by Alamar Blue^®^) in contact with different concentrations of MSN and MSN@PEI loaded or not with pleiotrophin (PTN) or in the presence of 0.05 nM exogenous PTN alone at 24 and 48 h of cell culture. Data are mean ± SEM of three independent experiments performed in triplicate. * *p* < 0.05 vs. hMSC and MSN@PEI 100 µg/mL; # *p* < 0.01 vs. PTN 0.05 nM and all groups.

**Figure 8:**
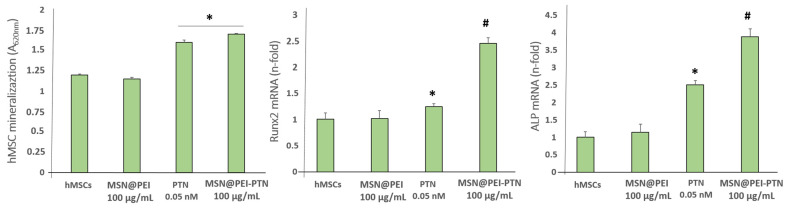
Mineralization and changes in osteoblastic gene expression in human Mesenchymal Stem Cells (hMSC) induced by different nanosystems loaded or not with pleiotrophin (PTN) or in the presence of 0.05 nM exogenous PTN alone. Mineralization was assessed using alizarin-red staining at day 15. The gene expression of Runx2 and ALP markers (analyzed by real time PCR) was measured at day 5. Data are mean ± SEM of three independent experiments performed in triplicate. * *p* < 0.05 vs. MSN 100 µg/mL; # *p* < 0.01 vs. PTN 0.05 nM and all groups.

The viability results of hMSCS were slightly different from those obtained in MC3T3-E1 cells. The best viability of hMSCs cells was observed when using MSN@PEI, with the best concentration being 100 μg/mL MSNs (Figure 7). However, a PEI coating slightly affected viability, although this effect disappeared over time. This fact could be explained by the mesenchymal nature of the cells, where the effects tend to be stronger due to their greater reactivity to stimuli. However, the presence of PEI at the surface of the MSNs did not drastically affect the cell viability in comparison with the hMSC control (Figure 7). As was the case with the MC3T3-E1 cells, the MSN@PEI nanocarrier at a concentration of 100 µg/mL was chosen for the rest of the experiments (Figure 7 and Figure 8) based on the internalization and viability results.

Very impressive results in human mesenchymal-cell viability were observed when loading PTN into the nanosystem. MSN@PEI-PTN nanosystems significantly increased viability over free PTN at a 0.05 nM concentration. A remarkable increase was observed in the first 24 h, indicating that the protein might be released. Additionally, loading PTN to our nanoplatform almost doubled hMSC viability, confirming the very positive effect of the nanocarrier here-designed in human bone cells.

The differentiation assays revealed an increase in the mineralization of hMSCs exposed to MSN@PEI-PTN and PTN, as previously observed in pre-osteoblastic cells. These results confirmed that the MSN@PEI-PTN system was as effective as the free protein alone (Figure 8). The consistency of this effect with results similar to those obtained in MC3T3-E1 cells confirms the efficiency of the nanoplatform proposed here independently of the cell evaluated. In addition, the complete nanocarrier with PEI and PTN improved the gene expression of Runx2 and ALP compared to that in free PTN. As observed in the mineralization, the results obtained in the gene expression of these osteoblastic markers in hMSCs were in agreement with those of mouse pre-osteoblastic cells, which confirms again that the nanosystem proposed here would trigger bone-tissue regeneration regardless of the cells line evaluated.

In addition, it is significant to note that the expression levels were higher than in the case of pre-osteoblastic cells. These results are in agreement with previous data that demonstrate that PTN transactivates endothelial-growth-factor receptor-activating Erk and Akt pathways in the primary osteoblast, which leads to an increase in ALP activity [45]. However, loading PTN into MSNs has doubled that positive effect on early bone-regeneration gene expression, which means that the platform developed here for the first time could be part of a new and effective treatment for bone regeneration.

## 4. Conclusions

The present proof-of-concept showed, for the first time, that PTN-protein loaded into mesoporous silica nanoparticles improved the viability and the osteogenic and differentiating actions in the absence of osteogenic differentiation-promoting medium in different cell lines. The here-developed nanoplatform was evaluated in two bone related cell lines, pre-osteoblastic cells and human mesenchymal stem cells, showing that the promising results in terms of viability and bone regeneration were independent of the in vitro model employed. The synthesis, functionalization, and PTN loading have been optimized to develop an effective pleiotrophin nanocarrier for the first time. The use of this novel nanoplatform increased the cell viability in comparison with free PTN and, more importantly, doubled the expression of certain genes indicative of the early bone-regeneration process. These findings could be of great importance for future developments of nanosystems for the potential treatment of osteoporosis or other bone-related diseases where fast and effective bone regeneration might be required.

## Data Availability

Not applicable.
